# Long Non-Coding RNAs: The Regulatory Mechanisms, Research Strategies, and Future Directions in Cancers

**DOI:** 10.3389/fonc.2020.598817

**Published:** 2020-12-18

**Authors:** Na Gao, Yueheng Li, Jing Li, Zhengfan Gao, Zhenzhen Yang, Yong Li, Hongtao Liu, Tianli Fan

**Affiliations:** ^1^ Department of Pharmacology, School of Basic Medicine, Zhengzhou University, Zhengzhou, China; ^2^ Translational Medicine Research Center, People’s Hospital of Zhengzhou, Zhengzhou, China; ^3^ Faculty of Medicine, St George and Sutherland Clinical School, St George Hospital, The University of New South Wales (UNSW) Sydney, Kensington, NSW, Australia; ^4^ Laboratory for Cell Biology, College of Life Sciences of Zhengzhou University, Zhengzhou, China

**Keywords:** long non-coding RNA, mechanism of action, research strategies, therapeutic targets, cancer

## Abstract

The development and application of whole genome sequencing technology has greatly broadened our horizons on the capabilities of long non-coding RNAs (lncRNAs). LncRNAs are more than 200 nucleotides in length and lack protein-coding potential. Increasing evidence indicates that lncRNAs exert an irreplaceable role in tumor initiation, progression, as well as metastasis, and are novel molecular biomarkers for diagnosis and prognosis of cancer patients. Furthermore, lncRNAs and the pathways they influence might represent promising therapeutic targets for a number of tumors. Here, we discuss the recent advances in understanding of the specific regulatory mechanisms of lncRNAs. We focused on the signal, decoy, guide, and scaffold functions of lncRNAs at the epigenetic, transcription, and post-transcription levels in cancer cells. Additionally, we summarize the research strategies used to investigate the roles of lncRNAs in tumors, including lncRNAs screening, lncRNAs characteristic analyses, functional studies, and molecular mechanisms of lncRNAs. This review will provide a short but comprehensive description of the lncRNA functions in tumor development and progression, thus accelerating the clinical implementation of lncRNAs as tumor biomarkers and therapeutic targets.

## Introduction

Cancer is a complex disease associated with multiple genetic mutations. Over the past few decades, oncogenes such as Src and Ras have been discovered, and the functions of their encoded proteins have been elucidated. While cancer awareness is gradually increasing worldwide, the current focus is still concentrated on the DNA sequence responsible for the abnormal protein synthesis. With the widespread application of next-generation sequencing (NGS) technology, a large number of non-coding genes have been identified and found to be strongly associated with tumor development and progression. Therefore, it is of paramount importance to understand the underlying mechanism through which non-coding genes influence the tumorigenic process.

In 1961, the central position of RNA in the flow of genetic information was revealed (1) and in the following 50 years, the emergence of whole-genome sequencing technology has greatly accelerated our understanding of both coding and non-coding RNAs (ncRNAs) (2, 3). Many regulatory RNAs harboring various sizes have been discovered (4), especially long non-coding RNAs (lncRNAs) (5). LncRNAs are a class of RNA molecules comprised of more than 200 nucleotides, which do not encode proteins. LncRNAs were originally thought to be by-products transcribed by RNA polymerase II and have no biological function; however, with the development of high-throughput sequencing technology, an increasing number of lncRNAs have been annotated, and their functions of lncRNAs in tumorigenesiss and tumor progression have been gradually elucidated. Previous studies showed that lncRNAs are involved in the regulation of cell survival, growth (6–10), invasion, and metastasis (11), maintenance of stemness (12, 13), as well as tumor angiogenesis (14). These studies highlight the essential role of lncRNAs in cancer development and progression, as well as their potential as novel therapeutic targets for multiple tumors.

LncRNAs are structurally similar to mRNAs and are also generated through DNA transcription (15). Based on the chromosomal position of the lncRNAs, they are divided into antisense lncRNAs, intronic lncRNAs, divergent lncRNAs, intergenic lncRNAs, promoter-associated lncRNAs, transcription start site-associated lncRNAs, and enhancer RNAs (eRNAs) (16–20). Interestingly, the number of lncRNAs far exceeds the number of protein-coding genes (21). Numerous lncRNAs are abnormally expressed in specific cancer types (22) and participate in a variety of complex biological processes by interacting with proteins, DNA, as well as RNAs (23–26). However, the specific mechanisms of abnormally expressed lncRNAs in cancer cells remain unclear. There are still many outstanding questions that need to be explored. For example, what are the specific modes of action of lncRNAs? How dose the location of lncRNAs in the nucleus or cytoplasm affect their mechanism of action? How can the research strategies of lncRNAs in cancer cells be explored?

Here, we describe the specific regulatory mechanisms of lncRNAs, mainly focusing on their signal, decoy, guide, and scaffold functions in cancer cells. The lncRNAs in the nucleus are mainly involved in epigenetic and transcriptional regulation while the lncRNAs in the cytoplasm are often involved in post-transcriptional regulation, thus regulating the mRNA stability, protein translation and the competitive endogenous RNA (ceRNA) network. In addition, the research strategies used to identify the roles of lncRNAs in tumors are summarized, including lncRNA screening, lncRNA characteristic analyses, functional studies and molecular mechanisms of lncRNAs. The current review will provide a comprehensive description of the lncRNA functions and novel insights into their underlying molecular mechanisms.

## The Mechanisms of Action of lncRNAs in Cancer

**Figure 1:**
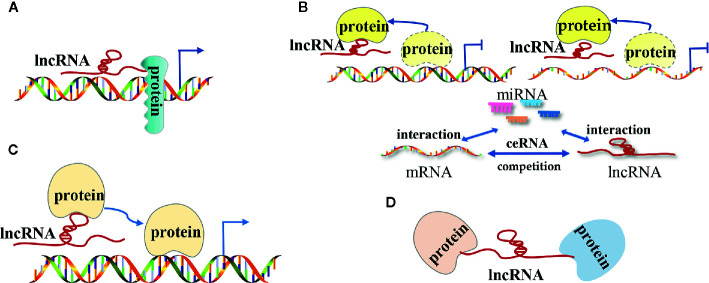
The modes of action of long non-coding RNAs (lncRNAs) in tumors. **(A)** LncRNAs as signal molecules can be used alone or combined with some proteins(such as transcription factors) to mediate the transcription of downstream genes; **(B)** LncRNAs as decoy molecules bind to some functional protein molecules to block the protein molecules from regulating DNA and mRNA molecules or bind to miRNA molecules competitively with mRNA molecules to block the inhibitory effect of miRNA on mRNA molecules; **(C)** LncRNAs as guide molecules carries some functional protein molecules and locates them in the target area to perform functions; **(D)** LncRNAs as a scaffold molecule guide related different types of macromolecular complexes to assemble in the target area to work together.

The lncRNA mechanisms of action can be divided into four categories: signal, decoy, guide, and scaffold (19, 27–29) (**Figure 1**).

### LncRNAs as Signal Molecules

As signal molecules, lncRNAs are often considered to regulate the transcription of downstream genes. Previous studies have demonstrated that lncRNAs are specifically transcribed and they influence certain signaling pathways under different stimulation conditions. The transcribed lncRNAs act by themselves or in combination with certein proteins (such as transcription factors) to mediate the transcription of downstream genes. Huarte et al. showed that p53-induced lncRNA-p21 interacted with nuclear heterogeneous ribonucleoprotein-K to inhibit the expression of downstream genes in the p53 signaling pathway (10). LncRNA PANDA, which is activated by the interaction between p53 and cyclin-dependent kinase inhibitor 1A (CDKN1A, p21) in the presence of DNA damage, prevents the expression of apoptosis-related genes by interacting with the nuclear transcription factor Y subunit α (NF-YA) to prolong the survival time of tumor cells (30). Nevertheless, the use of RNA for transcriptional regulation is homeostatically advantageous due to its capacity to influence protein as a rapid response to external factors that interact with the body.

### LncRNAs as Decoy Molecules

LncRNA commonly act as a decoy molecule by blocking a certain molecular pathway. After lncRNA is transcribed, it directly binds to some protein molecules such as chromosome folding proteins or transcription regulators and impairs the function of that protein. LncRNAs directly bind to transcription regulators to block the function of transcription factors, further suppressing the downstream gene transcription. For instance, DNA damage-induced lncRNA PANDA directly binds to the nuclear transcription factor NF-YA and impairs its function, thus preventing the expression of apoptosis-related genes and ultimately inhibiting the NF-YA-dependent apoptosis pathways (30). Furthermore, lncRNAs can interact with certain proteins in order to hinder the proteins’ capacity to regulate mRNA expression. For example, the lncRNA MALAT1 located in the nucleus regulates the phosphorylation status of serine/arginine (SR) by binding to the SR splicing factors that regulate the variable splicing of pre-mRNA (31).

LncRNAs can also affect the expression of target genes by sponging miRNAs. In certain tumor cells and specific tissues, lncRNAs directly bind to the miRNA molecules and prevent them binding to the target mRNAs, thus upregulating of the expression of the target genes. In prostate cancer, lncRNA PCAT-1 acts as a sponge for absorbs miR-3667-3p and reduce its inhibitory effect on c-Myc mRNA, which promotes the proliferation and migration of the tumor (32).

### LncRNAs as Guide Molecules

LncRNAs can act as guiding molecules by aiding specific protein in reaching their target location and exerting their biological functions. As guiding molecules, lncRNAs are often interacting with transcription factors, which are located on a specific DNA sequence and regulate gene transcription. Numerous studies showed that lncRNAs can regulate gene transcription through cis regulation, thus regulating the transcription of adjacent mRNAs. Wang et al. found that lncTCF7 can recruit the SWI/SNF complex to the TCF7 promoter and regulate the expression of TCF7. This process can trigger the activation of the Wnt signaling pathway, and ultimately promote the self-renewal of liver cancer stem cells and the proliferation of cancer cells (33). Nevertheless, lncRNAs can also regulate gene expression through trans regulation, which is characterized by lncRNAs’ capacity to regulate the transcription of remote mRNAs. For instance, lncRNA HOTAIR interacts with the polycomb repressive complex 2 (PRC2) and induces the relocation of PRC2 complex throughout the genome, thus promoting histone methylation modifications in several target genes (34). Tsai et al. showed that the two ends of the lncRNA HOTAIR interact with two different histone modification complexes (35). The 5′-domain of lncRNA HOTAIR binds to the PRC2 complex (methylation effect), while the 3′-domain binds to the LSD1/CoREST/REST complex (demethylation effect). Thus, these two complexes allow heterogeneous histone modification enzyme assembly. Lastly, the assembled histone modification enzymes localize to different gene regions and regulate the histone methylation patterns of several genes, thereby regulating the transcription of several target genes.

### LncRNAs as Scaffold Molecules

LncRNA can act as a “central platform” and facilitate the interaction of numerous molecules and protein. Furthermore, the scaffold properties of lncRNAs enable the assembly of different types of macromolecular complexes, thus promoting the convergence and integration of information among different signaling pathways (20, 36). One of the most important scaffold lncRNAs is the X-inactive speciﬁc transcript (Xist) RNA, which has a length of 17kb and is encoded by the X chromosome to be suppressed in females. Xist recruits the polycomb repressive complex 1 (PRC1) and PRC2 complexes, which suppress the gene expression of one X chromosome in females, thus having a dose compensation role in mammals (37, 38). Emerging evidence also confirmed that Xist RNA is associated with cancer (39). For instance, Xist acts as an oncogene in non-small cell lung cancer by recruiting EZH2 to the epigenetically repressed kruppel-like factor 2 gene (KLF2) (40). In addition to Xist, there are other lncRNAs that have scaffold regulation functions. LncRNA HOTAIR promotes the recruitment and interaction between lysine (K)-specific demethylase 1A (LSD1) and hepatitis B X-interacting protein (HBXIP), thus mediating the activation of transcription factor c-Myc, along with transcription activation of Cyclin A, elF4E, and LDHA (41). LncRNA INK4 is an antisense transcription product of cyclin-dependent kinase inhibitor 2B (CDKN2B, p15 INK4b), which can act as a scaffold for PRC1 and PRC2 complexes and result in the silencing of the tumor suppressor gene cyclin-dependent kinase inhibitor 2A (CDKN2A, p16 INK4a) (42). As a scaffold molecule, lncRNA CCAT1 was shown to bind two distinct epigenetic modification complexes [5’-domain of CCAT1 binds PRC2, while 3’-domain binds the suppressor of variegation 3-9 homolog 1 (SUV39H1)], thus regulating the histone methylation pattern of the sprouty RTK signaling antagonist 4 (SPRY4) promoter, and ultimately promoting the proliferation and metastasis of esophageal squamous cell carcinoma (ESCC) (43).

## LncRNAs Regulate Tumor Progression at Three Different Levels

**Figure 2:**
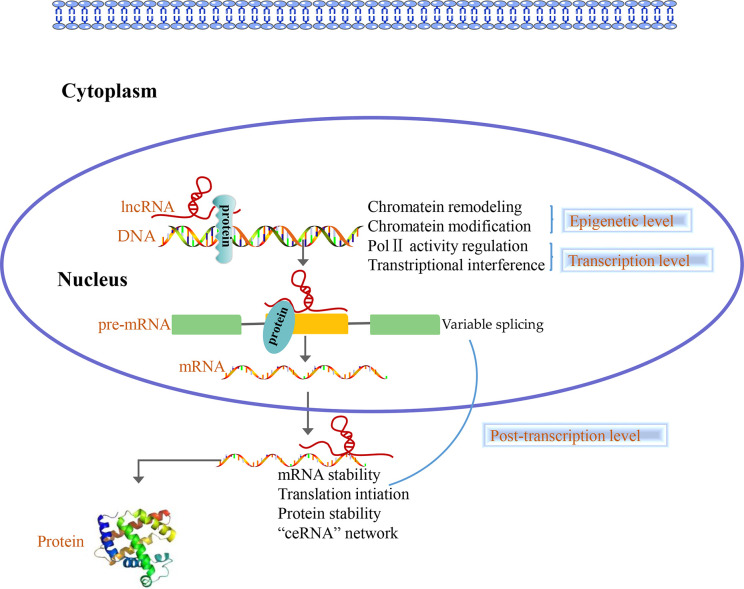
Long non-coding RNAs (LncRNAs) function at three levels. In the nucleus, lncRNAs control the epigenetic state of genes, participate in transcriptional regulation and are involved in the process of pre-mRNA alternative splicing. In the cytoplasm, lncRNAs are involved in post-transcriptional regulation such as the mRNAs stability, mRNAs translation, protein stability and “ceRNA” network.

Accumulating evidence shows that lncRNAs play different roles in the nucleus and cytoplasm. In the nucleus, lncRNAs regulate the epigenome by recruiting chromatin remodeling complexes as well as chromatin modification complexes. Furthermore, lncRNAs act as transcriptional regulators by themselves or by recruiting transcription factors and are involved in the process of pre-mRNA alternative splicing. Nevertheless, numerous studies indicated that there are a large number of lncRNAs in the cytoplasm, which are involved in post-transcriptional regulation processes such as the mRNAs stability, mRNAs translation, protein stability, and “ceRNA” network (44, 45) (**Figure 2**). Overall, lncRNAs can regulate the gene expression at epigenetic, transcriptional and post-transcriptional level.

### LncRNAs Participate in Epigenetic Regulation of Genes

Some lncRNAs manipulate gene expression by recruiting chromatin remodeling complexes and chromatin modification complexes to specific sites and influence the chromosome structure, histone modification status, and DNA methylation status (46, 47).

A recent report indicated that lncRNAs are associated with chromatin remodeling (48), and lncRNAs can recruit chromatin remodeling complexes, change chromatin structure, and regulate oncogene expression. Tang et al. demonstrated that after the interaction of lncRNAs with the chromatin remodeling complex switching defective/sucrose non-fermenting (SWI/SNF), the ATP hydrolysis energy was used to change the chromatin structure and regulate gene transcription, ultimately resulting in oncogene expression changes (49). It has been reported that lncRNA HOTAIR interacts with SMARCB1 and ARID1, which are subunits of the chromatin remodeling complex SWI/SNF and change the chromatin structure as well as promote the transcription of the SNAIL gene, ultimately promoting kidney cancer progression (50).

LncRNA-mediated changes in histone status are associated with H3K4me3, H3K9me2, and H3K27me3 modifications of the promoter region. These histone modifications change the chromatin structure and alter the expression of the underlying genes (51). The lncRNA HOTAIR transcribed by the HOXC gene cluster is one of most common lncRNAs that influence gene expression through histone modifications. The lncRNA HOTAIR can recruit the chromatin modification complex and locate it to the target gene region, thuschanging the chromatin state and regulating the transcription of several target genes (34, 35). In addition to lncRNA HOTAIR, there are other lncRNAs that recruit histone modification complexes to modify histone epigenetic patterns. for instance, lncRNA INK4 can act as a scaffold molecule, which promotes the interaction between PRC1 and PRC2 complexes, leading to histone modifications and silencing of the CDKN2A gene (42). LncRNA CCAT1 binds to PCR2 and SUV39H1 in order to regulate the histone methylation of the SPRY4 promoter region, thus promoting the proliferation and metastasis of ESCC (43). Furthermore, lncRNA HOXA11-AS binds to EZH2 to promote histone methylation of the p21 promoter region, thus inhibiting the transcription of the tumor suppressor gene p21 (52).

While lncRNAs can aid the localization of DNA methylases or demethylases to a specific target gene promoter, the DNA methylation is a dynamic and reversible process. Arab et al. showed that after lncRNA TARID binds to growth arrest and DNA-damage-inducible, alpha (GADD45A, with demethylation), the 5-methylcytosine on the TCF21 promoter (tumor suppressor) was demethylated, thereby mediating the transcriptional activation of TCF21 (53).

### LncRNAs Are Involved in Gene Transcriptional Regulation

In eukaryotic cells, transcription factors bind to DNA and control the transcription, localization, and stability of the RNA transcribed. Some lncRNAs that act as ligands often interact with transcription factors to form complexes and control gene transcription (54). LncRNAs regulate genetic transcription through cis and trans regulation. LncRNAs regulate the transcription expression of nearby mRNAs through cis regulation. To reveal if lncRNAs use cis regulation to influence gene transcription, first, we must observe the expression of nearby mRNAs following knockdown or overexpression of lncRNAs to confirm if there is a correlation between lncRNA and nearby mRNA levels. Next, we would have to determine whether the lncRNA recruits some proteins or protein complexes, which are attached to the promoter region of the target genes to achieve regulatory functions. For instance, Wang et al. found that lncTCF7 recruited SWI/SNF complex to the TCF7 promoter to regulate the expression of TCF7, thereby mediating the activation of the Wnt signaling pathway, and ultimately promoting the self-renewal of liver CSCs and tumor proliferation (33). However, if the expression of nearby functional genes does not change after lncRNAs are knocked down or overexpressed, lncRNAs are considered to regulate the transcription of genes through the trans mode of action. To reveal if lncRNAs use trans regulation to influence gene transcription, first, we should screen the proteins that might bind to the lncRNAs through experiments and bioinformatics, identify the potential target mRNAs, and analyze the correlation between the expression of lncRNAs and the target genes. Ultimately, we would have to prove that the lncRNA and its associated proteins bind to the target gene promoter region to regulate its transcription. Li et al. showed that lncRNA AGAP2-AS1 recruited EZH2 and LSD1 to the promoter regions of KLF2 and large tumor suppressor 2 (LATS2), thus, inhibiting the transcription of KLF2 as well as LATS2 and promoting the non-small cell lung cancer progression (55). Certain lncRNAs act as transcription factors themselves. Ketab et al. discovered that lncRNA GAS5 folds into a DNA-like structure that binds to the glucocorticoid receptor (GR), and inhibits GR transcription activity. Finally, lncRNA GAS5 can reduce the production of red blood cells, platelets, and white blood cells, which was associated with to a poorer prognosis for acute myeloid leukemia (56).

### LncRNAs Control Gene Expression by Post-Transcriptional Level

In addition to the two regulatory mechanisms aforementioned, lncRNAs are involved in post-transcriptional regulation, including alternative splicing of pre-mRNA, stabilization of mRNA, and translation and stabilization of proteins (57–59). Most lncRNAs involved in the post-transcriptional regulation of mRNAs are antisense lncRNAs. In the pre-mRNA variable splicing regulation process, antisense lncRNAs act by themselves or combined with splicing factors to control the pre-mRNA splicing process. For instance, lnc-Spry1 binds to U2 small nuclear ribonucleoprotein auxiliary factor 65kD splicing factor, and influences the variable splicing process of fibroblast growth factor receptor pre-mRNA related to the epithelial-mesenchymal transition (EMT) (60). Furthermore, lncRNAs can regulate mRNA stability. For instance, the lncRNA PXN-AS1 can be expressed as the PXN-AS1-L (large) or PXN-AS1-S (small) transcript. Compared to PXN-AS1-S, PXN-AS1-Lhas an extra exon. Due to the presence of the exon 4 sequence, PXN-AS1-L binds to the 3’UTR region of the Paxillin (PXN) mRNA, which impairs the binding of miRNA-24 to the PXN mRNA and reduces its degradation (61). LncRNAs also regulate protein translation. For instance, lncRNA GAS5 recruits the translation initiation factor eIF4E and allows it to bind to the c-Myc mRNA, thus inhibiting c-Myc protein translation and ultimately downregulating the c-Myc expression (62). LncRNA MT1JP directly binds to cytotoxic granule-associated RNA binding protein-like 1 (TIAR) to enhance the translation process of p53 mRNA, thereby up-regulating p53 expression (63). LncRNAs also regulate protein stability. For example, lncRNA UPAT interacts with ubiquitin-like with PHD and RING finger domains 1 (UHRF1) protein to ensure its stability by interfering with its ubiquitination process (64).

LncRNAs often affect the expression of their target genes by interacting with miRNAs, which are the main post-transcriptional regulation factors. In some tumor cells and specific tissues, some lncRNAs carrying “seed sequences” of certain miRNAs bind to miRNAs and act like sponges, thereby preventing miRNAs from binding to their target mRNAs (65–67). Qu et al. showed that lncARSR promotes the expression of Anexelekto and cellular-mesenchymal to epithelial transition factor in renal cancer cells through competitive binding to miR-34/miR-449, thereby increasing the resistance to sunitinib (68). Jia et al. revealed that lncRNA H19 acts as a sponge for miR-29a, thus upregulating angiogenesis factor vasohibin 2 and promoting angiogenesis of glioma and other biological processes of endothelial cells associated with glioma (69). Zhang et al. showed that lncRNA CCAT1 promoted the expression of the transcription factor homeobox gene B1 by sponging miR-7 from the cytoplasm, thereby promoting the proliferation and metastasis of ESCC (43).

In summary, it has been found that lncRNAs regulate gene expression at following three levels by interfering with the epigenome and by regulating the transcriptional as well as post-transcriptional processes of the targeted genes.

## Research Strategies of lncRNAs in Tumor

**Figure 3:**
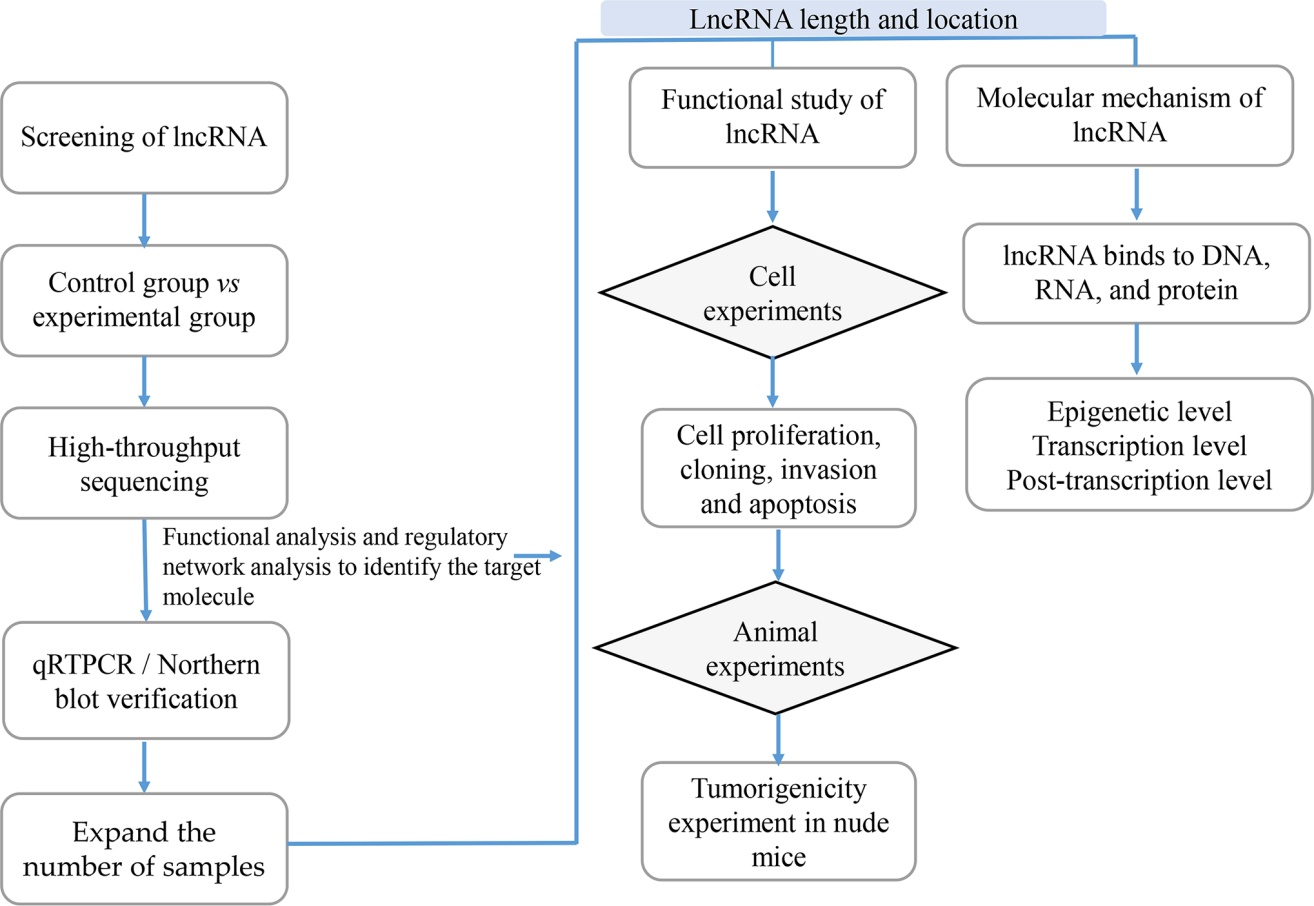
The Research Strategies of Long Non-Coding RNAs (lncRNAs) in Tumors. In order to study lncRNAs, high-throughput sequencing technology or high-throughput data in the database are usually used to screen out candidate lncRNAs, while qRTPCR and Northern/blot technologies are used for verification; then RACE and FISH technologies are used to determine the full length and location of lncRNAs; the functional research of lncRNAs is usually verified from *in vitro* experiments including cell proliferation, cloning, invasion and apoptosis, and *in vivo* experiments mainly including tumor-bearing experiments in nude mice; the research on the mechanism of lncRNAs is usually carried out at the epigenetic level, transcription level and post-transcriptional level according to their cellular location.

With the development of high-throughput sequencing technology, an increasing number of lncRNAs are annotated; however, the function of most lncRNAs in tumors remains unclear. Since the field of lncRNAs remains broadly unknown, its exploration is of paramount importance. Previous studies showed that the function of lncRNAs in tumors can be investigated using lncRNA screening, lncRNA characteristic analyses, functional studies and lncRNA molecular mechanisms analyses (**Figure 3**).

### Screening of lncRNAs

Under the premise of guaranteeing at least three samples, differentially expressed lncRNAs can be screened using high-throughput sequencing technologies such as NGS (70). Various tissues, whole blood, cells, plasma, serum, and exosomes can be used to screen the differentially expressed lncRNAs according to the purpose of the experiment. High-throughput sequencing technology is sensitive enough to detect rare transcripts that have only a few copies and can also detect unknown genes and new transcripts with a wide detection range. However, its high costs will limit its widespread application in different laboratories (71).

The lncRNAs of interest were also obtained from databases such as the TCGA database, a multi-omics database related to tumors, which includes DNA-Sep, RNA-Sep, protein-Sep, and other omics data (72).

Following lncRNA screening, qRT-PCR/northern blot were used to reduce the number of candidate lncRNAs and verify their expression in clinical specimens and cell lines. Furthermore, the correlation between the lncRNA expression and clinical indicators would be determined, thus identifying the importance of the candidate lncRNAs in clinical diagnosis and treatment.

### Characteristic Analysis of lncRNAs

The mostly explored characteristics of lncRNAs include coding potential, location information on the genome, secondary structure, correlation with disease, full-length analysis, and cell localization, which can be determined through bioinformatical analysis. NONCODE, LncBook, LncRNAdb v2.0, and LncRNADisease are some softwares that can be used to obtain a provide comprehensive annotations of lncRNAs (73–77).

Rapid-amplification of cDNA ends (RACE) is a method that can provide the full length of the lncRNAs by extending and amplifying the two ends of a known cDNA fragment based on PCR technology (78–80). Before constructing an overexpression plasmid, researchers often clone the full-length candidate lncRNA and identify its sequence through 5’-RACE and 3’-RACE (81).

Understanding the cellular localization of lncRNAs helps us to understand their potential molecular mechanisms. Fluorescence *in situ* hybridization (FISH) is a method often used to identify the cellular localization of the candidate lncRNAs. The principle is that the foreign nucleic acid containing radioactive labels (3H, 32P, 35S, 125I) or non-radioactive labels [biotin, digoxin, horseradish peroxidase, fluorescein (FITC, rhodamine)] is complementary paired with the DNA or RNA to be tested on tissues, cells or chromosomes to form a specific nucleic acid hybrid molecule (82, 83). In addition to FISH localization, certain prediction software such as LncATLAS (84) can predict the cellular localization of lncRNAs; however, the predictive data need to be verified experimentally.

### The Functional Study of lncRNAs

Before exploring the specific lncRNAs’ molecular mechanisms of action, their functions should be elucidated. The functions of lncRNAs are often verified through *in vitro* and *in vivo* experiments.

*In vitro* experiments: Following overexpression or knock down of certain lncRNAs, their role in tumor progression is analyzed by measuring the changes in biological behaviors, phenotype, EMT markers, stem cell markers, and drug sensitivity. *In vivo* experiments: First, a lentiviral expression vector capable to knock down or overexpress a specific lncRNA is constructed. Next, the virus is transfected into cells, which are first screened for resistance, and then analyzed using qRT-PCR to detect the knockdown or overexpression levels of the candidate lncRNAs. Finally, the stably transfected cell strains that were screened are inoculated into the backs of nude mice, and the tumorigenicity of the candidate lncRNAs is verified using the nude mouse tumorigenic model or other animal models (85).

### The Molecular Mechanism of lncRNAs

If the lncRNAs are localized in the nucleus, they are considered to play regulatory roles at the chromatin and transcription levels, while the lncRNAs are localized in the cytoplasm, are considered to play regulatory role at the post-transcriptional level.

#### LncRNAs Affect the Expression of Downstream Genes by Mediating Chromatin Remodeling and Chromatin Modification

LncRNAs regulate chromatin remodeling, DNA methylation, and histone modification by DNA methylase and histone modification enzymes. The cat RAPID database can evaluate the protein-RNA binding tendency through the contribution of secondary structure, hydrogen bonding and van der Waals forces, thus it can provide an accurate prediction of the protein-lncRNA binding ability (86). The use of databases plays an auxiliary role in studying the interactions of lncRNAs with DNA methylases and histone modification enzymes. Generally, the epigenetic mechanisms of lncRNAs are confirmed through the following four aspects: (1) Confirmation of expression correlation. Following knock down or overexpression of lncRNAs, qRT-PCR, northern blot, and western blot should be used to observe whether the expression of the target mRNAs, histone modification enzymes and DNA methylases are affected; (2) Dissection of the chromatin status of the target genes. Following knock down or overexpression of lncRNAs, DNA-FISH experiments should be used to observe the effect of lncRNAs on the chromatin state of the target genes (87–89); (3) LncRNAs role in histone modification. Following knock down or overexpression of lncRNAs, chromatin immunoprecipitation (ChIP) (90, 91) and chromatin isolation by RNA purification (ChIRP) (92) experiments should be used to analyze the effect of lncRNAs on histone modification. The RNA-pull down and RNA immunoprecipitation (RIP) (92) experiments can provide information regarding the binding ability of lncRNAs to the histone modification enzyme; (4) The role of lncRNAs in the regulation of DNA methylation modification. Following knock down or overexpression of lncRNAs, methylation-specific PCR (MSP) and bisulfite sequencing PCR (BSP) (93, 94) experiments should be used to investigate the effects of lncRNAs on DNA methylation modification.

#### LncRNAs Can Regulate the Transcription of Target Genes Alone or in Combination With Transcription Factors

Whether lncRNAs regulate gene transcription through the cis or trans action mode depends on its relative position tothe target genes. Numerous databases provide valuable information regarding the combination of lncRNAs and transcription factors. For example, the ChIPBase v2.0 database integrates the binding sites of lncRNAs and transcription factors identified using the ChIP-Seq method (95). Generally, the influence of lncRNAs on transcriptional regulation of target genes was explored using the following processes: (1) Confirmation of expression correlation. Following knock down or overexpression of lncRNAs, qRT-PCR should be used to investigate the changes in target mRNA expression; (2) Whether lncRNAs recruit transcription regulators. Following lncRNA knock down or overexpression, RNA-pull down/RIP should be used to explore the binding ability of lncRNA and RNA-binding proteins; (3) Whether lncRNAs regulate the transcription of target genes: After lncRNA is knocked down or overexpressed, ChIP and ChIRP experiments should be used to analyze the regulation of lncRNAs on target gene transcription.

#### LncRNA as a ceRNA

The ceRNA network is one of main methods through which lncRNAs exert post-transcriptional regulation in the cytoplasm. Previous studies have shown that lncRNAs can competitively bind to miRNAs and affect the miRNA’ function on the target genes. The core experiments usually performed to prove the functions of ceRNA are: (1) Use of bioinformatics to predict the possible “ceRNA” networks. There are many databases that can predict possible “ceRNA” networks, such as Starbase (96), LncACTdb 2.0 (97), LncBase Predicted v2 (98), TargetScan (99), which contains all kinds of ceRNA regulatory relationships including miRNA-mRNA, miRNA-lncRNA, miRNA-circRNA and miRNA-ceRNA; (2) Confirmation of expression correlation. Following knock down or overexpression lncRNA, the changes in miRNA should be investigated; (3) Verifying the interaction of lncRNA, miRNA, and mRNA. Luciferase reporters should be used to confirm the interactions between lncRNAs, miRNAs, and mRNAs (100, 101).

In conclusion, the use of bioinformatics to predict the functions of lncRNAs is favored over the traditional, time-consuming, and expensive experimental methods. For the lncRNA field, it is essential to establish a variety of databases meant to help researchers identify and name their newly discovered lncRNAs. The already established databases for lncRNA are listed in (**Table 1**).

**Table 1:** Databases related to long non-coding RNA (lncRNA) research.

**Database**	**Internet site**	**Function**	**Literatures**
TCGA	https://www.cancer.gov/about-nci/organization/ccg/research/structural-genomics/tcga	Preliminary screening of lncRNA	(72)
NONCODE	http://www.noncode.org	Comprehensive annotation of lncRNA	(73–74)
LncBook	http://bigd.big.ac.cn/lncbook/index	Comprehensive annotation of lncRNA	(74–75)
lncRNAdb v2.0	http://lncrnadb.org	Comprehensive annotation of lncRNA	(76)
LncRNADisease	http://www.cuilab.cn/lncrnadisease	The relationship between lncRNA and disease	(77)
lncATLAS	http://lncatlas.crg.eu/	The cell localization of lncRNA	(84)
cat RAPID	http://service.tartaglialab.com/page/catrapid_group	Predict the combination of lncRNA and protein	(86)
ChIPBase v2.0	http://rna.sysu.edu.cn/chipbase/	Identify transcription factor binding sites mediated by lncRNA	(95)
Starbase	http://starbase.sysu.edu.cn/tutorialAPI.php	ceRNA	(96)
LncACTdb 2.0	http://www.bio-bigdata.net/LncACTdb/index.html	ceRNA	(97)
LncBase Predicted v2	http://carolina.imis.athena-innovation.gr/diana_tools/web/index.php?r=lncbasev2/index-predicted	ceRNA	(98)
TargetScan	http://www.targetscan.org/vert_72/	ceRNA	(99)

## Conclusions

The role of lncRNAs has become one ofmain focus for fundamental and clinical tumor studies. Given the increasing pool of evidence regarding the role of lncRNAs in tumor development and progression, here we summarized the four main models of action through which lncRNAs influence tumor progression (as a signal, decoy, guide, or scaffold molecule). The lncRNAs in the nucleus are often involved in chromatin remodeling and modification, transcriptional regulation, and alternative splicing of pre-mRNA, while the lncRNAs in the cytoplasm are often involved in the stability of mRNAs, protein translation, and the ceRNA network (102). Based on previous studies, we also propose the specific research strategies through which the lncRNA functions can be investigated (lncRNA screening, lncRNA characteristic analyses, functional studies, and molecular mechanisms of lncRNAs).

**Figure 4:**
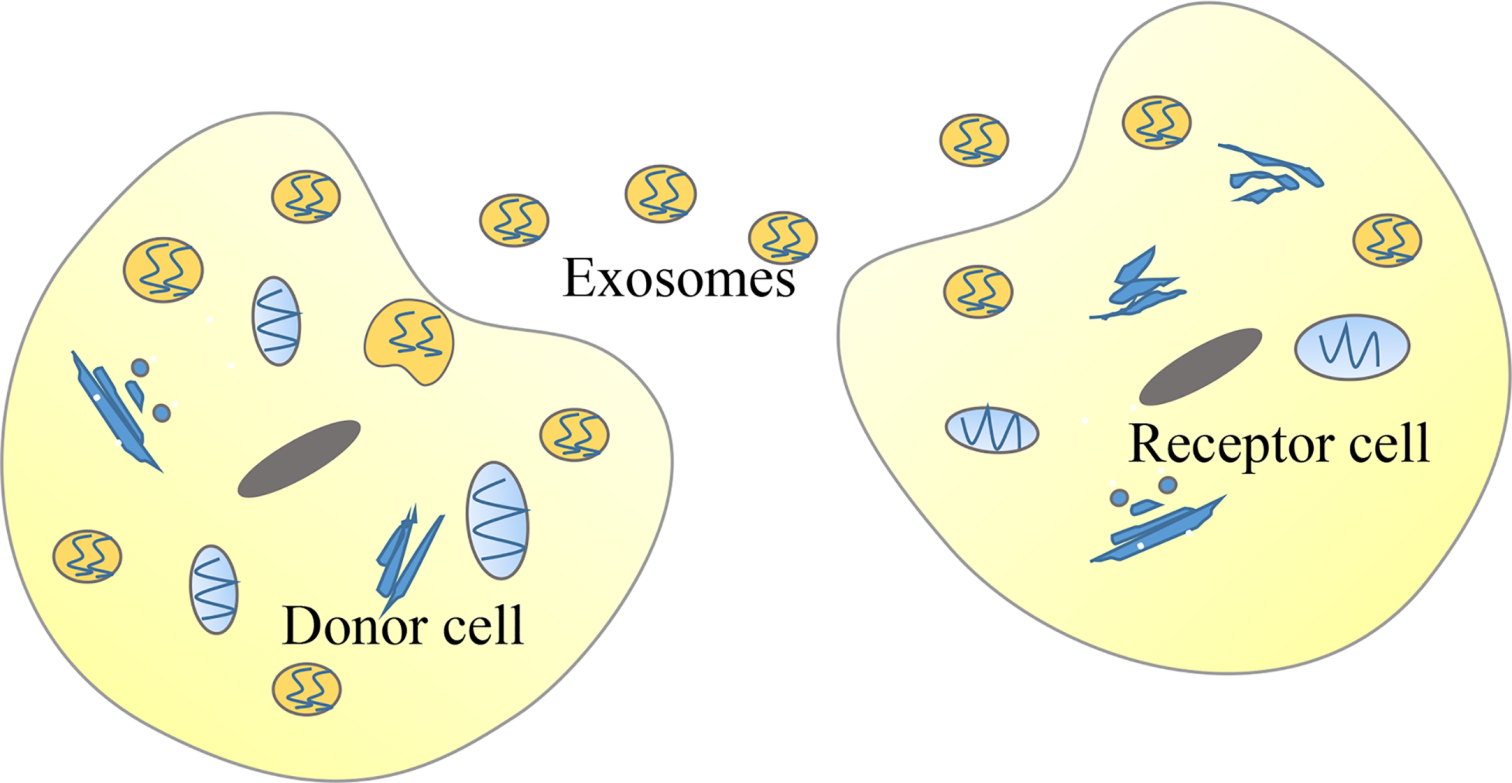
Tumor cells can deliver long non-coding RNAs (lncRNAs) through exosomes. In cancer, donor cells can deliver exosomes-encapsulated lncRNAs to recipient cells so that lncRNAs function in recipient cells.

Meanwhile, these findings raise further questions. For example: what is the mechanism that causes the abnormal expression of lncRNAs in tumors? Many upstream regulatory mechanisms of lncRNAs have not been elucidated. A potential new direction for future research would be to investigate the upstream regulatory factors of lncRNAs. According to previous literature, the expression of lncRNAs may be regulated by histone status, DNA methylation patterns, transcription factors, and post-transcriptional regulation. For example, the activation mechanism of lncRNA CCAT1, which can promote the proliferation and metastasis of ESCC, is represented by the acetylation of histone H3K27 (43). Moreover, the high expression of lncRNA H19 is due to the decreased methylation level of the CpG islands in the promoter region (103). Additionally, the high expression of lnc01503, which can promote the progression of ESCC, is due to the capacity of the transcription factor to bind to the promoter region (104). Lastly, the interaction between IGF2BP1 and the lncRNA HULC reduces the stability of lncRNA HULC and decreases its expression (105). Numerous types of lncRNAs have been classified according to their position relative to the genome. Regardless of their similarities, their mechanism of action is not exactly the same. The effects of eRNAs on the formation and stabilization of the chromatin loop between the enhancer and the promoter and their capacity to regulate the expression of some target genes at close and long distances became highly investigated recently (106, 107). Notably, previous studies have reported that eRNAs are a subclass of lncRNAs that play a critical role in cancer development (108). For example, HPSE eRNA promotes cancer progression by interfering with the chromatin looping and regulating the hnRNPU/p300/EGR1/HPSE axis (109). Based on the vast number of eRNAs and their expression regulation to some target genes without distance and cell type limitation, eRNAs may become potential targets for the diagnosis and treatment of human cancers (110, 111). Liquid biopsies based on exosome contents represent a potential direction for future molecular diagnosis as well as evaluation of chemotherapy effects and cancer prognosis, which have great application values for early detection of disease (112–118). Recent studies have shown that some lncRNAs can be encapsulated in exosomes, and exosomes can be used as a medium to transmit lncRNAs among tumor cells, thereby regulating the occurrence and development of tumors (119–123) (**Figure 4**). For example, lncARSR delivered *via* exosomes can promote sunitinib resistance as a competitive endogenous RNA in renal cancer (68). Furthermore, lncRNA PART1 delivered *via* exosomes can induce gefitinib resistance as a competitive endogenous RNA in ESCC (124). Lastly, chimeric RNA GOLM1-NAA35 from salivary exosomes might represent a potential biomarker for esophageal cancer (125). This would allow us to understand specific pathological conditions in cancer patients through the detection of specific lncRNAs encapsulated by exosomes (126). A previous study showed that engineered exosomes with phospholipid bilayer structures can be used to load certain anti-tumor drugs, therapeutic miRNAs, or proteins and target tumor cells (127).

In conclusion, the lncRNAs field is highly investigated due to their potential key role in cancer development and progression. A comprehensive understanding of lncRNAs in cancer signaling will stimulate new directions for future research, diagnosis, and therapies (128). LncRNAs are of great significance for the early diagnosis of cancer patients due to their abnormal expression changes as the cancer progresses. For example, the lncRNA prostate cancer antigen 3, which has been studied for early diagnosis, is a prostate-specific lncRNA, which can be detected with high specificity and sensitivity (129). Loewen et al. discussed the potential of lncRNA HOTAIR in the diagnosis and treatment of lung cancer (130). In addation, lncRNAs also serve as promising therapeutic targets. Given their divers modes of action, lncRNAs can be targeted using multiple approaches: (1) Regulate lncRNA genes through spatial blockade of promoters or the use of genome editing technology; (2) Regulate lncRNA levels through siRNAs; (3) Prevent lncRNAs from exerting their effect by inhibiting the interaction between RNAs and proteins or prevent the formation of secondary structures (131, 132). Recently, an increasing number of researchers considered lncRNAs as potential therapeutic targets for cancer. For example, Shin et al. discussed the feasibility of lncRNA BC200 as a cancer therapeutic target (133). Liu et al. investigated whether lncRNA PANDAR is a powerful diagnostic and therapeutic marker for patients with gastric cancer (134). The effective application of lncRNAs as potential targets for diagnosis and therapy has broad prospects for future cancer treatment. However, there are still numerous limitations for the use of lncRNAs as potential therapeutic targets and biomarker development. Nevertheless, with the rapid development of biochemical toolkits and technological breakthroughs for lncRNA research, an increasing number of lncRNAs are being discovered. However, the increasing rate of discovery of new lncRNAs presents challenges to their definition and annotation, which requires more comprehensive transcriptome analyses and transcription assembly. In addition, the functional characterization of lncRNAs is still challenging, mainly due to the complexity and diversity of their association with cancer cell functions. Overall, elucidating the mechanism of abnormal expression of lncRNAs and the downstream mechanism of lncRNAs in tumors would allow us to better understand lncRNAs and provide novel tumor markers for clinical diagnosis and prognostic evaluation of tumors.

## Author Contributions

NG, YHL, JL, ZG, and ZY wrote the manuscript, which TF, HL, and YL revised. All authors contributed to the article and approved the submitted version.

## Funding

This work was supported by the Key Scientific Research Projects of Colleges and Universities in Henan Province (No.20A310021 TF).

## Conflict of Interest

The authors declare that the research was conducted in the absence of any commercial or financial relationships that could be construed as a potential conflict of interest.
